# Advances and Challenges in Understanding Atmospheric Oxidizing Capacity in China: Insights from Chemical Mechanisms and Model Applications

**DOI:** 10.3390/toxics14020159

**Published:** 2026-02-08

**Authors:** Peixuan Li, Yanqin Ren, Fang Bi, Fangyun Long, Junling Li, Haijie Zhang, Zhenhai Wu, Hong Li

**Affiliations:** State Key Laboratory of Environmental Criteria and Risk Assessment, Chinese Research Academy of Environmental Sciences, Beijing 100012, China; lipeixuan24@mails.ucas.ac.cn (P.L.); longfangyun22@mails.ucas.ac.cn (F.L.); lijl@craes.org.cn (J.L.); zhanghaijie@craes.org.cn (H.Z.); wuzh01@craes.org.cn (Z.W.); lihong@craes.org.cn (H.L.)

**Keywords:** atmospheric oxidizing capacity, Secondary Organic Aerosol (SOA), Volatile Organic Compounds (VOCs), chemical transport model, radical chemistry

## Abstract

The ability of the atmosphere to convert primary pollutants into secondary pollutants through atmospheric oxidants is referred to as the atmospheric oxidizing capacity (AOC). This study systematically reviews the generation mechanisms, influencing factors, and quantitative characterization methods of major oxidants, along with advances in chemical mechanisms and modeling. We provide a comparative analysis of AOCs across diverse environments, including urban, suburban, and rural regions, highlighting the distinct impacts of anthropogenic and biogenic emissions on oxidation regimes. Despite advancements in chemical transport models and machine learning, limitations such as sparse observations, imperfect parameterizations, and unresolved chemical mechanisms lead to significant underestimations of the AOC. Future research must prioritize multi-scale observational networks and the elucidation of key chemical processes to refine model accuracy and improve the effectiveness of pollution control strategies.

## 1. Introduction

The atmospheric oxidizing capacity (AOC), defined as the integrated capability of the atmosphere to transform primary pollutants into secondary species via oxidants, serves as the fundamental driver of PM_2.5_-O_3_ co-pollution [1]. The formation processes of secondary particulate matter (PM) and ozone (O_3_) share common chemical pathways, both of which are critically dependent on radical-initiated oxidation of primary pollutants [2]. Accelerated urbanization and industrialization have increased anthropogenic emissions of volatile organic compounds (VOCs) and nitrogen oxides (NO_x_) substantially, thereby enhancing the AOC and consequently exacerbating secondary organic aerosol (SOA) formation and surface-level O_3_ pollution [2]. Chronic exposure to elevated O_3_ and PM_2.5_ (wherein SOA constitutes a major component) is robustly associated with increased risks of respiratory diseases and cardiovascular mortality [3]. Moreover, SOA significantly influences regional climate through solar radiation scattering and absorption [4]. Consequently, understanding the spatiotemporal evolution and regulatory mechanisms of AOC is paramount for both air quality improvement and climate change mitigation.

Significant advances have recently been made in elucidating oxidant production mechanisms, characterizing regional AOC variations, and refining modeling techniques. A notable divergence exists in global trends: while global nighttime AOC has generally declined, in China, it exhibits a significant enhancement [5]. The accelerated NO_3_-mediated nocturnal oxidation within China shortens the lifetime of NO_x_, promotes nitrate aerosol formation, and intensifies PM_2.5_-O_3_ co-pollution. Although the implementation of China’s clean air initiatives (e.g., the Air Pollution Prevention and Control Action Plan, initiated in 2013) has substantially reduced anthropogenic emissions (PM_2.5_, NO_x_, SO_2_), VOC levels have remained largely unchanged. This unbalanced emission reduction has paradoxically enhanced the AOC, elevating concentrations of key oxidants and radicals (O_3_ and RO_x_ [=OH+HO_2_+RO_2_]) [6]. Consequently, while secondary inorganic aerosols (e.g., sulfate, nitrate) have decreased, SOA’s contribution to PM_2.5_ has increased [7]. This is particularly evident during autumn haze events in Beijing, where observed O_3_ enhancement correlates strongly with elevated SOA contributions [8].

Importantly, the AOC exhibits a threshold effect governing secondary aerosol formation: secondary aerosol production dominates when the maximum daily 8 h average ozone concentration (MDA8 O_3_) ≤ 100 μg m^−3^, whereas primary emissions prevail at concentrations >160 μg m^−3^ [9]. This phenomenon highlights the limitations of conventional primary pollution control strategies and underscores the imperative for synergistic approaches targeting AOC modulation through coordinated NO_x_-VOC control. This review systematically synthesizes recent advances in AOC research, focusing on (1) key chemical processes and influencing factors, (2) spatiotemporal patterns across diverse environments (urban, suburban, rural), and (3) progress in chemical mechanisms and model optimization. By identifying critical knowledge gaps and proposing targeted recommendations, this work provides theoretical support for developing “AOC-specific” pollution control strategies.

## 2. Characterization of Atmospheric Oxidizing Capacity and Its Key Chemical Processes

### 2.1. Characterization

Photolysis of O_3_, a key oxidant, generates highly reactive O(^1^D) atoms that initiate VOC oxidation chains. Meanwhile, hydroxyl (OH) and nitrate (NO_3_) radicals dominate daytime and nighttime oxidation processes, respectively [10,11]. The AOC serves as a fundamental driver for the formation of secondary pollutants such as O_3_ and SOA in the troposphere [1,2,12]. It is most typically quantified by the concentration of oxidants (O_x_ = O_3_ + NO_2_) or the aggregate rate of oxidation reactions [13], and most studies solely take OH oxidation into account when computing OH reactivity, which represents the overall rate of oxidation reactions [14]. The total OH reactivity (k_OH_) characterizes the number of molecules that are consumed by OH radicals per unit time and per unit volume, i.e., the instantaneous net rate of loss of OH radicals, with the dimension of s^−1^ [15,16]. By definition, OH reactivity is limited to a subset of oxidation pathways. Its measurement is technically demanding, and its values can fluctuate sharply near pollution sources owing to the influence of short-lived VOCs and CO. These characteristics diminish its reliability for assessing regional background levels or long-term trends. O_x_ specifically refers to the sum of O_3_ and NO_2_, which is a concentration metric under photochemical steady-state conditions that is capable of eliminating the influence of NO-O_3_ titration and more stably reflecting the total amount of oxidants that are generated by atmospheric photochemistry [17,18]. However, it does not encompass other critical atmospheric oxidants such as OH, RO_2_, and NO_3_, which may lead to an underestimation of the total oxidative capacity under certain conditions.

To address the challenge of quantifying the AOC, Liu et al. innovatively proposed a dual-index framework: the Apparent Oxidative Index (AOI_e_) evaluates oxidation from a product perspective, while the Atmospheric Oxidative Potential Index (AOI_p_) quantifies the consumption rates of primary precursors by oxidants [19]. The study established mathematical formulations and closure methods for both indices, achieving for the first time a normalized quantitative characterization of AOC in complex urban environments. Application of this method to the Beijing and Xianghe regions revealed that current mainstream atmospheric chemical mechanisms systematically underestimate the actual AOC [19,20].

The theoretical foundation is rooted in the electron transfer processes during oxidation of primary pollutants (NO_x_, SO_2_, VOCs) to secondary species (NO_3_^−^, SO_4_^2−^, oxygenated organic compounds) by atmospheric oxidants (OH, O_3_, NO_3_). AOI_e_ and AOI_p_ quantify the AOC from thermodynamic and kinetic perspectives, respectively.

Apparent Oxidative Index (electron-transfer-based metric):

$${AOI}_{e}=f_{e1({NO}_{x}\;to\;{NO}_{3}^{-})}+f_{e2({SO}_{2}\;to\;SO_{4}^{2-})}+f_{e3(VOCs\;to\;SOA)}+f_{e4(NO\;to\;NO_{2})}+{\;f}_{e5(O\;to\;O_{3})}$$

where *fe*_1_–*fe*_5_ represents molar electron transfer during oxidation processes (e.g., *fe*_4_ = 2 × [NO_2_]).

The SOA contribution (*fe*_3_) is calculated as follows:

$$f_{e3(VOCs\;to\;SOA)}=SOA\times \left(\frac{O/C}{1+O/C}\right)\times 2$$

Oxidative Potential Index (reaction-kinetics-based metric):

$${AOI}_{p}=\sum_{j=1}^{m}\left\{\left[C_{j}\right]\times \sum_{i=1}^{n}k_{ij}\left[X_{i}\right]\right\}$$

where [*C_j_*] denotes the concentration of precursors (including VOCs, CO, NO_x_, and SO_2_), [*X_i_*] represents the concentrations of oxidants (OH, O_3_, NO_3_), and *k_ij_* is the bimolecular rate constant for reactions between *C_j_* and *X_i_*.

AOI_e_ and AOI_p_ are novel metrics designed to quantify the AOC. AOI_e_ is strongly dependent on the concentrations of long-lived secondary pollutants that are generated via local transformation and regional transport, making it more indicative of regional-scale AOC than local AOC. However, AOI_e_ may underestimate the AOC by neglecting heterogeneous oxidation pathways in SOA formation, while the inter-regional transport and background accumulation of secondary pollutants can lead to its overestimation. The calculation of AOI_p_ relies on the accuracy of reaction rate constants and the concentrations of radicals (such as OH and NO_3_). If the reaction rate constants are inaccurate, the results for AOI_p_ will also be affected. These radical concentrations are typically estimated through parameterization methods, which carry a certain degree of uncertainty. The calculation of AOI_p_ only considers gas-phase reactions, neglecting the contributions of liquid-phase reactions and heterogeneous reactions to the AOC. This can lead to an underestimation of the AOC, especially in regions where liquid-phase and heterogeneous reactions play significant roles. By incorporating model-simulated OVOC reactions and heterogeneous oxidation processes into the AOI_p_ calculation, the underestimation of the AOC by AOI_p_ is significantly improved.

### 2.2. Key Processes

**Figure 1:**
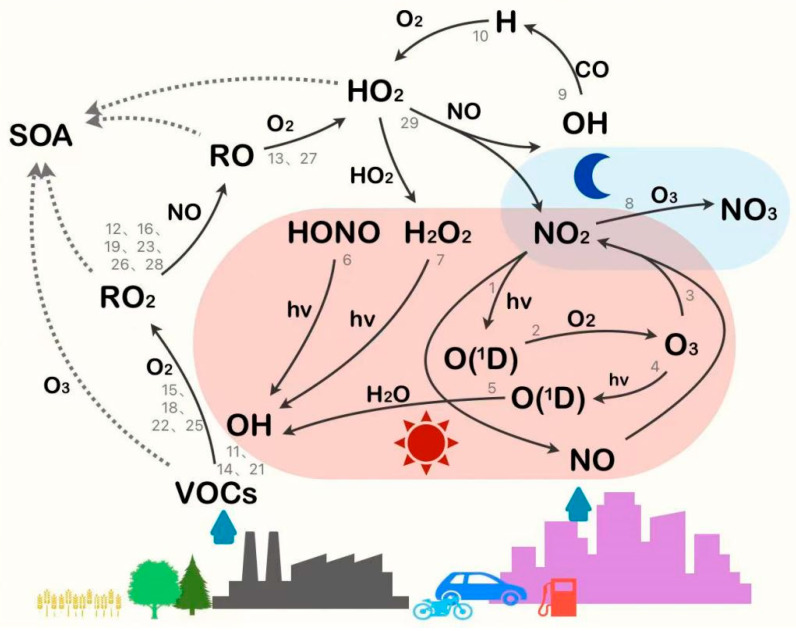
Schematic diagram of key reaction mechanisms governing atmospheric oxidizing capacity (the gray reaction numbers correspond to equations in text).

Photochemical oxidation processes play the vital role in shaping AOC during the warm seasons. This is clearly reflected in Beijing, where AOI_e_-based calculations show that gas-phase oxidation products (O_3_ and NO_2_) contribute over 80% to the total AOC in summer. Even in winter, when particle-phase products play an enhanced role, gas-phase products maintain their dominance, constituting about 70% of the AOC [19]. Friedlander and Seinfeld [21] first proposed the theoretical framework for photochemical smog formation (Table 1). The formation of tropospheric O_3_ involves a series of photochemical reactions, with VOCs and NO_x_ serving as key precursors, exhibiting complex nonlinear relationships with O_3_ concentrations [22,23]. As shown in Figure 1, beyond participating in O_3_ formation through reactions with NO_x_ under solar radiation, VOCs undergo oxidation by OH, O_3_, and NO_3_ radicals to form SOA, yielding various products, including peroxy radicals (RO_2_, HO_2_), carbonyl compounds (aldehydes, ketones), and organic peroxides. The fundamental steps of O_3_ photochemical production involve the following:

(1) NO2+hv→NO+OD1

(2) OD1+O2+M→O3+M

(3) $$O_{3}+NO\to NO_{2}+O_{2}$$

OH, the most reactive atmospheric oxidant, originates primarily from the following:

(4) O3+hvλ<320 nm→O2+OD1

(5) OD1+H2O→2OH

(6) $$HONO+hv\left(\lambda <400\;nm\right)\to OH+NO$$

(7) $$H_{2}O_{2}+hv\left(\lambda <360\;nm\right)\to 2OH$$

NO_3_, a dominant nighttime atmospheric oxidant, forms as follows:

(8) $${NO}_{2}+O_{3}\to {NO}_{3}+O_{2}$$

OH, as the most reactive atmospheric oxidant, plays a central role in oxidizing most major atmospheric pollutants [24,25,26]. For instance, it reacts with carbon monoxide (CO) to form hydroperoxyl radicals (HO_2_) (Reactions 9–10) and with VOCs to generate organic peroxy radicals (RO_2_) (Reactions 11, 14, 21, 15, 18, 22, 25). Subsequently, RO_2_ reacts with nitric oxide (NO) to form alkoxy radicals (RO) and nitrogen dioxide (NO_2_) (Reactions 12, 16, 19, 23, 26, 28). The unstable RO radicals undergo hydrogen abstraction and further react with oxygen (O_2_) to produce HO_2_ (Reactions 13 and 27). HO_2_ can then react with NO, regenerating OH and NO_2_ (Reaction 29). This cyclic interconversion among OH, HO_2_, and RO_2_ drives the continuous conversion of NO to NO_2_, ultimately leading to net O_3_ production [27].

OH to HO_2_ conversion:

(9) $$OH+CO\to H+CO_{2}$$

(10) $$O_{2}+H\to HO_{2}$$

Alkane (C_n_H_2n+2_) oxidation:

(11) $$C_{4}H_{10}+OH\to C_{4}H_{9}O_{2}+H_{2}O$$

(12) $$C_{4}H_{9}O_{2}+NO\to C_{4}H_{9}O+NO_{2}$$

(13) $$C_{4}H_{9}O+O_{2}\to C_{3}H_{7}CHO+HO_{2}$$

Alkene (C_n_H_2n_) oxidation:

(14) $$C_{4}H_{8}+OH\to C_{4}H_{8}-OH$$

(15) $$HOC_{4}H_{8}+O_{2}\to HOC_{4}H_{8}O_{2}$$

(16) $$HOC_{4}H_{8}O_{2}+NO\to HOC_{4}H_{8}O+NO_{2}$$

(17) $$HOC_{4}H_{8}O\to HOCH_{2}+{\left(CH_{3}\right)}_{2}CO$$

(18) $$HOCH_{2}+O_{2}\to HOCH_{2}O_{2}$$

(19) $$HOCH_{2}O_{2}+NO\to HOCH_{2}O+NO_{2}$$

(20) $$HOCH_{2}O\to CH_{2}O+OH$$

Aldehyde (R-CHO) oxidation:

(21) $$CH_{3}CHO+OH\to CH_{3}CO+H_{2}O$$

(22) $$CH_{3}CO+O_{2}\to CH_{3}C\left(O\right)O_{2}$$

(23) $$CH_{3}C\left(O\right)O_{2}+NO\to CH_{3}C\left(O\right)O+NO_{2}$$

(24) $$CH_{3}C\left(O\right)O\to CH_{3}+CO_{2}$$

(25) $$CH_{3}+O_{2}\to CH_{3}O_{2}$$

(26) $$CH_{3}O_{2}+NO\to CH_{3}O+NO_{2}$$

(27) $$CH_{3}O+O_{2}\to HCHO+HO_{2}$$

These peroxy radicals subsequently participate in chain-propagating reactions, sustaining the NO-to-NO_2_ conversion cycle that drives net O_3_ production:

(28) $$RO_{2}+NO\to RO+NO_{2}$$

(29) $$HO_{2}+NO\to OH+NO_{2}$$

VOCs, encompassing both biogenic and anthropogenic sources (e.g., industrial emissions, vehicle exhaust, and solvent use), are crucial components in tropospheric chemistry, as they are primary precursors for both O_3_ and SOA formation [28,29,30]. As crucial intermediates in atmospheric oxidation reactions, RO_2_ radicals play a pivotal role in the process of PM_2.5_-O_3_ compound pollution. The complex reaction networks involving RO_2_, NO_x_, and HO_x_ directly regulate the production levels of O_3_ and SOA. The formation of SOA is significantly influenced by NO_x_ concentration through its modulation of RO_2_ radical reaction pathways. Under high-NO_x_ conditions, the reaction pathway of RO_2_ with NO_x_ is dominant. This not only suppresses the RO_2_ autoxidation process but also tends to yield more volatile products, leading to a reduction in SOA yield [31]. In contrast, under low-NO_x_ conditions, RO_2_ primarily reacts with HO_2_ or other RO_2_ radicals, favoring the production of low-volatility products and thereby increasing SOA yield [32]. Upon initiation by atmospheric oxidants such as OH radicals, VOCs undergo a sequence of radical-chain oxidation steps. In this process, the radical center propagates through RO_2_- or RO-mediated isomerization, occasionally accompanied by R rearrangement, until chain termination occurs, yielding closed-shell products [33]. Concurrently, the resulting RO_2_ radicals can rapidly form highly oxygenated organic molecules (HOMs) via intramolecular H-shift and subsequent O_2_ addition, a pathway recognized as RO_2_ autoxidation, which substantially promotes SOA formation [34]. Since the reaction mechanisms of RO_2_ radicals are intrinsically linked to their specific structure (e.g., isomeric form), this structure-reactivity relationship has motivated sustained efforts to develop analytical methods for the real-time, isomer-specific detection of these transient species, which is inherently difficult due to the vast diversity and structural complexity in the RO_2_ family [35]. Studies were conducted to identify the isomers and rotamers of propyl peroxy radical [35] and ethyl peroxy radical [36] utilizing the technique of vacuum ultraviolet (VUV) synchrotron radiation spectroscopy combined accurate theoretical computations. Li et al. [37] investigated the atmospheric oxidation mechanism of cyclohexene, focusing on RO_2_ formation and transformation. Using proton transfer reaction mass spectrometry for direct measurement of RO_2_ and closed-shell products, combined with box model simulations, their work revealed the limitations in current mechanisms regarding RO_2_ autoxidation and OH-initiated oxidation pathways.

In recent decades, various atmospheric chemistry mechanisms have been developed to formalize complex atmospheric chemical processes through mathematical modeling, enabling quantitative simulation and objective description of the transformation of primary pollutants into secondary pollutants [38]. A thorough understanding of these mechanisms is crucial for elucidating pollution formation and developing effective control strategies.

Atmospheric chemistry mechanisms can be classified into detailed and lumped mechanisms [39]. Both types describe the chemistry of VOCs and inorganic species (O_x_, HO_x_, NO_x_, and SO_x_). The Master Chemical Mechanism (MCM) is a widely used detailed gas-phase mechanism that explicitly describes the reaction pathways of individual VOC species without lumping simplifications [38,40]. It is suitable for assessing O_3_ formation potential and VOC transformation, SOA precursor analysis, and cloud chemistry studies, helping reveal variations in atmospheric oxidizing capacity under different conditions. However, its high computational cost limits its integration with regional models.

In contrast, lumped mechanisms such as the Carbon Bond Mechanism (CBM), Statewide Air Pollution Research Center (SAPRC) mechanism, Regional Acid Deposition Model (RADM), and Regional Atmospheric Chemistry Mechanism (RACM) employ VOC classification and lumping techniques. The CBM, SAPRC, and RADM are often applied in regional air quality models [38], while the MCM and RACM are primarily used in box model studies [40]. These mechanisms have been validated through smog chamber experiments and are advantageous for simulating the characteristics of VOC pollution, estimating O_3_ production rates, and studying SOA formation. The CBM excels in simulating regional pollution with simplified organic chemistry, the SAPRC accurately represents real-world VOC pollution, and the RACM details the photochemical generation and radical chemistry of O_3_.

### 2.3. Key Influencing Factors Other than Anthropogenic VOCs

Substantial previous research has confirmed that anthropogenic volatile organic compounds (AVOCs) represent a major factor influencing the intensity of the AOC [41,42,43]. Herein, we summarize several other key factors beyond AVOCs, including meteorological parameters, aerosol effects, and biogenic volatile organic compound (BVOC) emissions. Meteorological parameters such as solar radiation critically regulate the pathways and rates of atmospheric photochemical reactions, directly influencing oxidant concentrations and the kinetics of oxidation processes. Atmospheric aerosols can significantly modify the AOC and influence SOA formation through direct and indirect radiative effects. BVOCs contribute significantly to the formation of atmospheric oxidants (e.g., O_3_) and exhibit substantial Secondary Organic Aerosol Formation Potential (SOAFP).

#### 2.3.1. Meteorological Influences

Photolysis reactions account for 58% to 86% of primary radical sources during daytime [14]. Solar radiation, as the primary driver of photochemical reactions, plays a pivotal role in modulating atmospheric pollutants and oxidants. In China, between 2013 and 2017, variations in meteorological factors (including temperature and radiation) drove changes exceeding 20% in MDA8 O_3_ levels under temperature fluctuations within 1 °C [44]. Bei et al. employed the WRF-Chem model to investigate aerosol–radiation interaction (ARI) under varying synoptic conditions during winter in the Guanzhong Basin [45]. Their results demonstrated that ARI reduces the solar radiation intensity and surface temperature while suppressing boundary layer development, contributing to 15–25% of near-surface PM_2.5_ concentrations. Temperature and circulation accounted for approximately 37% of the increase in O_3_ levels in China between 2013 and 2017 [46].

Stagnant wind conditions hinder the transport, dilution, and dispersion of air pollutants. Such pollution episodes are often associated with valley-basin topography under the influence of large-scale high-pressure ridges [9,47]. Wind speed serves as a critical meteorological factor governing regional pollutant dispersion and transport, typically exhibiting a strong negative correlation with aerosol concentrations. Humidity plays a key role in heterogeneous nucleation of aerosols and hygroscopic growth processes, particularly in the aqueous-phase oxidation of SO_2_. When the relative humidity (RH) exceeds 70%, the probability of high-concentration pollution events involving submicron particulate matter (PM_1_) increases significantly. The boundary layer height (BLH) represents another crucial factor affecting the dispersion or accumulation of tropospheric pollutants. A shallow boundary layer restricts vertical and horizontal aerosol diffusion, leading to pollutant accumulation and exacerbating air pollution [48].

#### 2.3.2. Aerosol Effects

Atmospheric aerosols influence the AOC through three primary mechanisms: First, their direct radiative effects often lead to negative radiative forcing (the “umbrella effect”) via the absorption and scattering of solar radiation, which modifies temperature, humidity, and wind patterns, ultimately influencing photochemical reaction rates. Second, aerosols interact with clouds by serving as cloud condensation nuclei (CCN) and ice nuclei (IN), indirectly altering atmospheric oxidizability through changes in cloud microphysics and precipitation processes. Third, they facilitate heterogeneous chemistry by providing reactive surfaces that drive the production and consumption of key oxidants. Together, these mechanisms highlight the complex and multifaceted role of aerosols in shaping atmospheric chemical reactivity. Case studies in the Yangtze River Delta reveal that black carbon (BC) reduces surface O_3_ concentrations by 8–12% through light absorption, with the magnitude depending on the BC mixing state and size distribution [49]. These effects can substantially modify Empirical Kinetic Modeling Approach (EKMA) curves, potentially shifting O_3_ production regimes.

#### 2.3.3. Biogenic VOC Emissions

In addition to anthropogenic sources, biogenic emissions also constitute a significant contributor to VOCs in atmosphere [29,50]. During summer in Los Angeles, BVOC emissions account for approximately 60% of the hydroxyl reactivity (OHR) and SOAFP, with this contribution rate increasing significantly with rising temperatures [51]. The annual total BVOC emissions in the Beijing–Tianjin–Hebei (BTH) region in 2018 were estimated via two emission inventory methods, the biomass-based method and the Model of Emissions of Gases and Aerosols from Nature (MEGAN), yielding values of 734.6 Gg y^−1^ and 777.3 Gg y^−1^, respectively. Regarding the compositional profile of BVOC emissions in this region, the average contribution ratios of isoprene, monoterpenes, and other VOCs were 36.86%, 28.52%, and 34.62%, respectively. Spatially, the areas with high BVOC emissions were primarily concentrated in the central and northern mountains of the BTH region. The ozone formation potential (OFP) and SOA formation potential (SOAFP) were calculated to quantify the role of BVOCs in driving O_3_ and SOA formation. Simulation results indicated that BVOC emissions contributed an average of 23.73% to the surface O_3_ concentrations and 37.99% to the SOA concentrations in July across 13 cities in the BTH region [30]. This significant impact is primarily due to the surge in BVOC emissions (especially highly reactive isoprene and monoterpenes) under high summer temperatures, which affects downwind cities under the influence of regional transport. Among the O_3_ that is generated by BVOCs, crops contribute the most, accounting for 46.3%; deciduous forests are the second largest contributor, at 27.34%; and the vegetation type with the smallest contribution is fruiters, at only 4.98%. Of the SOA that is generated by BVOCs, crops are again the largest contributor, reaching as high as 56.63%; deciduous forests contribute 17.73%; and evergreen forests and shrubs and grasslands make relatively smaller contributions, both below 10%. BVOC emissions from different vegetation types exhibit varying contributions to O_3_ and SOA formation. BVOCs that are emitted by crops contain a higher proportion of highly reactive isoprene and monoterpenes, which can produce more O_3_ and SOA per unit mass. Coniferous forests typically emit higher proportions of monoterpenes, whereas broadleaf forests release more isoprene. Deciduous forests exhibit greater influence on O_3_ concentrations than evergreen forests, with distinct contribution patterns for SOA concentrations. The contribution of deciduous forests to BVOCs-O_3_ (27.34%) is significantly higher than their contribution to BSOA (17.73%), which is consistent with their characteristic of primarily emitting isoprene. In the Beijing–Tianjin–Hebei region, deciduous species such as poplar and Quercus contribute most significantly to O_3_ concentrations, while Pinus tabuliformis and Quercus play dominant roles in SOA formation [30]. It should be noted that these conclusions are based on simulations in summer. BVOC emissions are highly dependent on temperature and light, with summer being the peak emission period. Therefore, these contribution rates only represent summer levels and may not be applicable to O_3_ pollution events in autumn or winter.

## 3. The Regional Characteristics of the AOC

Driven by solar radiation, the AOC exhibits pronounced diurnal variations, with the daytime intensity generally surpassing nighttime levels. Under illuminated conditions, photochemical reactions dominate the production of oxidants such as O_3_ and facilitate the conversion of primary organic aerosols (POA) to SOA. O_3_, OH, and NO_x_ constitute three key atmospheric oxidants, which are ubiquitously present in urban, suburban, and rural environments (Table S1). In urban and suburban areas dominated by traffic and industrial emissions, as well as in rural regions with significant biogenic emissions, OH serves as the primary daytime atmospheric oxidant. During nighttime, the contributions of O_3_ and NO_x_ become more prominent. O_3_ production is nonlinearly regulated by its precursors, VOCs and NO_x_, which lead to two distinct control regimes: the NO_x_-limited regime and the VOC-limited regime. In the NO_x_-limited regime, O_3_ formation is primarily governed by NO_x_ concentrations, where NO_x_ reduction effectively lowers O_3_ levels, while changes in VOC concentrations exhibit minimal impact. The opposite holds true for the VOC-limited regime. The Beijing–Tianjin–Hebei urban agglomeration and its surrounding suburbs predominantly fall within the VOC-limited regime. In the low-VOC-concentration scenario, the reaction system is within the VOC-limited regime. As NO_x_ decreases and the VOC/NO_x_ ratio increases, the system transitions from the VOC-limited regime to the NO_x_-limited regime, with the O_3_ concentration first rising and then declining. Correspondingly, the yields of SOA and the concentration of OH also increase initially before decreasing [52]. In the NO_x_-limited regime under high VOC concentrations, reducing NO_x_ simultaneously suppresses the formation of O_3_ and SOA, which is related to the decline in the system’s oxidation capacity: decreased NO_x_ leads to reduced OH concentrations, thereby inhibiting the production of low-volatility oxygenated/nitrogenated oxidation products [52,53].

Through the synthesis of atmospheric oxidant and pollutant concentration data across different environments, distinct disparities emerge in pollution characteristics and oxidative properties among urban, suburban, and rural areas. Urban regions, characterized by high-density traffic and residential activities, exhibit elevated pollutant and atmospheric oxidant concentrations. Suburban areas, influenced by urban expansion, experience a combination of transported and locally emitted pollution, resulting in intermediate air pollution levels and atmospheric oxidant concentrations between those of urban and rural settings. In contrast, rural areas generally feature lower pollution levels, although agricultural activities and seasonal factors may still induce localized pollution, primarily driven by primary emissions (e.g., fossil fuel and biomass burning). Emissions of BVOCs from vegetation significantly contribute to the formation of SOA in rural areas. With ongoing urban expansion, rising concentrations of NO_x_ in some rural regions are increasingly participating in the atmospheric oxidation processes that form secondary pollutants. This phenomenon leads to elevated ozone levels in rural environments and enhances SOA formation.

This section highlights the spatial and temporal heterogeneity of the AOC, emphasizing the need for region-specific pollution control strategies to mitigate oxidative pollution effectively. The AOC patterns elucidated in this study capture oxidative chemistry across diverse atmospheric settings, including both densely populated urban areas that are influenced strongly by anthropogenic emissions and regions characterized by mixed anthropogenic and biogenic sources. Consequently, direct extrapolation of these AOC patterns to other geographical or climatic contexts should be approached with caution, as the AOC’s magnitude and sensitivity are modulated by factors that vary substantially across spaces and seasons, such as VOC speciation, NO_x_ regimes, background aerosol composition, and actinic flux. Therefore, while the AOC regimes and responses characterized here are representative of environments with similar emission profiles and chemical conditions, the underlying chemical mechanisms identified—notably key RO_2_ isomerization pathways and NO_x_-VOC coupling—offer transferable insights that can inform model development.

In this study, the identification of VOC-limited or NO_x_-limited regimes is primarily based on the EKMA from specific observation periods and existing regional modeling results. It should be noted that such classifications are strongly influenced by the accuracy of VOC/NO_x_ input data, the adopted chemical reaction mechanisms, and the actual meteorological conditions. Therefore, they should be regarded as preliminary diagnoses of the dominant regime in a given period or region, rather than definitive conclusions. Moreover, the findings of this study reflect more on the potential spatial patterns of the AOC and its macro-scale influencing factors, whereas precise quantification of absolute inter-regional differences would require the establishment of a unified, long-term, high-resolution, and vertically resolved observational network in the future.

### 3.1. Urban Areas

Elevated concentrations of NO_x_ and VOCs in urban areas enhance the O_3_ formation potential substantially. The photochemical production of O_3_ exhibits a complex nonlinear response to its precursors [27]. Based on WRF-Chem simulations and EKMA analysis, the Beijing urban area is identified as a VOC-limited regime in summer, characterized by the following key features: (1) VOC reduction leads to a pronounced decrease in O_3_ concentrations; (2) concurrent VOC increase and NO_x_ reduction synergistically elevate O_3_ levels; (3) NO_x_ increase suppresses O_3_ production by consuming OH; and (4) vertically, the O_3_ concentration gradient weakens with altitude, accompanied by a reduction in the VOC-limited regime and enhanced NO_x_ sensitivity [54].

Previous studies have quantified atmospheric oxidative capacity in Chinese urban areas and highlighted the dominant role of specific oxidants across different times of day and pollution conditions. For instance, Shao et al. [55] quantified the AOC in the urban area of Taiyuan during summer using the AOI_p_. The results indicated that OH radicals dominated daytime oxidation under both clean and polluted conditions, contributing 97.7–97.8% of the total AOC. The AOC level during the daytime was dozens of times higher than that at night. Similarly, Jia et al. [12] employed a comprehensive reaction rate index, which considers oxidants such as OH, O_3_, and NO_3_ and precursors including VOCs, CO, and CH_4_ to assess AOC in Beijing during autumn. The study showed that OH was the dominant oxidant during daytime, whereas O_3_, and NO_3_ jointly controlled the oxidation process at night. Specifically, during an O_3_ episode, the contributions of OH, O_3_, and NO_3_ to the total AOC were 87%, 10%, and 3% during the daytime, and 29%, 61%, and 10% at night, respectively; during a PM_2.5_ episode, the corresponding contributions were 86%, 6%, and 7% in the daytime, and 29%, 10%, and 61% at night. As demonstrated, OH was the dominant oxidant during the daytime in both episodes, accounting for approximately 90% of the total AOC. NO_3_ is an important nocturnal oxidant, and NO_3_ oxidation contributes an average of 10–20% to SOA formation. During the period from 2014 to 2019, China experienced a significant increase in the nitrate radical production rate (PNO_3_), with an average annual growth rate of 5.8%. This rise in PNO_3_ was primarily driven by the increase in nocturnal O_3_ concentrations. Regions exhibiting high nocturnal PNO_3_ were predominantly concentrated in the urban clusters of eastern China, such as the North China Plain and the Yangtze River Delta [5].

Based on intensive observations conducted in four major Chinese cities—Beijing (July), Shanghai (August), Guangzhou (October), and Chongqing (August)—the study by Tan et al. [14] systematically measured ozone, nitrogen oxides, CO, VOCs, photolysis frequencies, and meteorological parameters. It employed a box model with the RACM2 chemical mechanism, constrained by observations, to simulate the concentrations of OH, HO_2_, and RO_2_ radicals. Based on this foundation, a systematic analysis of the radical budget and quantitative calculations of the ozone formation potential were carried out. The findings reveal significant urban differences in OH reactivity, with Guangzhou exhibiting the highest levels (20–30 s^−1^) and Shanghai the lowest (<15 s^−1^). Over 50% of OH reactivity across these cities stems from inorganic compounds (CO and NO_x_). Simulated OH concentrations show a strong correlation with photolysis frequencies, peaking at approximately 7 × 10^6^ cm^−3^ in Beijing and Shanghai, followed by 4 × 10^6^ cm^−3^ in Chongqing, and the lowest at 2 × 10^6^ cm^−3^ in Guangzhou. In contrast, peroxyl radical concentrations reach their highest in Chongqing, with HO_2_ peaking at 5 × 10^8^ cm^−3^ and RO_2_ at 7 × 10^8^ cm^−3^, primarily attributed to its elevated VOC/NO_x_ ratio. Radical budget analysis identifies the photolysis of HONO, O_3_, and HCHO as the primary sources of radicals, with the decomposition of alkenes by ozone standing out in Guangzhou, contributing 43% to daytime primary sources. Regarding the formation mechanisms of secondary pollutants, the study reveals that the local ozone production rate is highest in Beijing (with a daily cumulative production of 136 ppbv) and lowest in Guangzhou (40 ppbv). All observed cities are in VOC-limited regimes. Relative incremental reactivity analysis further confirms that AVOCs are the most sensitive precursors for ozone formation. Concurrently, the study indicates that photochemically produced HNO_3_ serves as a crucial precursor for particulate nitrate under ammonia-rich conditions, highlighting the reduction in photochemical nitric acid formation as a key strategy for controlling summertime nitrate pollution. Overall, Beijing exhibits the strongest daytime OH oxidation rate (peaking at approximately 10 ppbv h^−1^), signifying its highest atmospheric oxidation capacity. This strong oxidizing capacity directly drives the formation of secondary pollutants such as ozone and nitrate. Nevertheless, this study has limitations in terms of the generalizability of its conclusions and mechanistic understanding. Firstly, the seasonal scope is restricted—observations were limited to a single month of peak photochemical pollution in each city—and the study is thus unable to capture seasonal variations in atmospheric oxidation mechanisms. For example, the potential shift from VOC-limited to NO_x_-limited ozone formation under winter conditions (low temperature, weak radiation) remains unverified. Secondly, uncertainties exist in characterizing key precursors: HONO concentrations were derived from a fixed ratio (2%) of NO_2_ rather than direct measurements, ignoring potential biases from spatiotemporal heterogeneity in HONO sources and sinks, despite limited sensitivity in model outputs (when HONO halves, OH changes <10%). Thirdly, inherent model deficiencies may affect its quantitative accuracy: under high-NO_x_ conditions, current mechanisms tend to underestimate HO_2_ and RO_2_ concentrations, leading to underestimated ozone production rates. Finally, the study’s spatial representativeness is limited, as conclusions are based on urban center measurements without fully addressing regional-scale pollutant transport and chemical evolution.

Based on a PM_2.5_-O_3_ compound pollution episode observed at an urban station in Beijing from 13 to 23 May 2017, Cui et al. conducted sensitivity experiments using the WRF-Chem model and EKMA curves. Compared with the scenario of increasing or decreasing VOCs or NO_x_ by 25% individually, their results demonstrate that simultaneous 25% reductions in VOCs and NO_x_ yield the most significant decrease in peak O_3_ concentrations of 43.3 μg/m^3^. However, a strategy of ~50% VOC reduction in urban areas was proposed to achieve compliance with the O_3_-1 h air quality standard [54]. In this case study, the ambient atmospheric condition (represented by 100% NO_x_ and 100% VOCs) is plotted within the VOC-limited quadrant of the EKMA isopleth diagram. This demonstrates that, given the prevailing NO_x_/VOC ratio, the O_3_ formation regime exhibits greater sensitivity to variations in VOC concentrations. Specifically, holding NO_x_ levels constant while increasing VOCs leads to a pronounced enhancement in O_3_ production; conversely, a reduction in VOCs results in a significant suppression of O_3_. Notably, the −25% NO_x_ perturbation scenario yields an O_3_ decrease of 29.4 μg m^−3^, a response magnitude that is comparable to the 32.9 μg m^−3^ reduction achieved under the −25% VOC scenario in the urban area. These comparable sensitivity indices suggest that the urban photochemical regime resides near the boundary between the VOC-limited and transitional regimes. Nevertheless, the overall O_3_ formation mechanism remains predominantly governed by the availability of VOCs, as indicated by the EKMA analysis. This conclusion is based on a specific process in May 2017 and may not apply to other seasons or years due to the strong nonlinearity of O_3_ generation. It should be noted that the model simulations contain inherent inaccuracies, for instance, the model results show a systematic underestimation of urban O_3_ concentrations, with a mean bias of −43.38 µg m^−3^ (NMB = −32.71%). Meanwhile, VOCs are simplified as a homogeneous component, without distinguishing the significant differences in reactivity among different species, which may affect the accurate quantification of the effects and thresholds of VOC emission reduction.

Kang et al. combined MAX-DOAS tower-based vertical observations with TROPOMI satellite vertical column density (VCD) data to retrieve the diurnal variation and vertical profile of NO_2_ concentrations in Beijing during spring 2019. Their results indicate that surface NO_2_ peaks in the early morning, reaches its minimum between 14:00 and 15:00 local time, and is strongly correlated with traffic emissions. Enhanced daytime turbulence facilitates NO_2_ transport from the boundary layer (BL) to the free troposphere (FT). In the evening, the BL height decreases, trapping free-tropospheric NO_2_ in the residual layer (RL). Morning planetary boundary layer (PBL) development triggers RL pollutant entrainment, resulting in higher NO_2_ concentrations in the RL than in the BL at 06:00 [56]. This study confirms that NO_2_ in Beijing’s urban atmosphere is predominantly derived from local emissions, and its concentration distribution is significantly constrained by the vertical structure of the PBL. A typical localized pollution pattern is observed, characterized by enrichment within the boundary layer and a sharp decline above it. This conclusion is supported by the strong correlations (with R values mostly exceeding 0.87) between MAX-DOAS observations and data from tower and satellite measurements, further emphasizing the dominant role of urban-scale anthropogenic emissions in shaping the near-surface spatial distribution of NO_2_.

### 3.2. Suburban Areas

Regarding vertical VOC distributions, Liu et al. collected atmospheric samples at altitudes of 50–400 m using drone-deployed stainless-steel canisters in Jinshan, a suburban area of Shanghai, during 8–9th September 2016. Fifty-two VOCs were quantified via gas chromatography–mass spectrometry (GC-MS), and their vertical profiles were investigated using principal component analysis (PCA). Vertically, the daily mean VOC concentrations increased from 50 to 100 m (peak concentration: 36.1 ppbv), remained elevated at 100–200 m (±5% variability), and decreased markedly above 200 m (21.2% lower at 400 m than at 100 m). With increasing altitudes, the fractions of alkanes and aromatics rose by 2.1% and 2.5%, respectively, while that of alkenes decreased by 4.8%. PCA identified the petrochemical industry, liquefied petroleum gas (LPG), and vehicular emissions as dominant VOC sources. Toluene and m/p-xylene were identified as key species for controlling near-surface O_3_ and SOA formation based on SOAFP and OFP calculations [22]. This study was the first to use drone technology to collect vertical distribution data of VOCs in the Yangtze River Delta region, revealing the concentration, composition, and chemical changes in VOCs at different heights, as well as their relationship with O_3_ and SOA formation. However, the study area is concentrated near petrochemical industrial and motor vehicle exhaust emission sources and does not consider the chemical reactions (e.g., oxidation) of VOCs during atmospheric transport. Thus, future research should consider integrating chemical reaction models to more comprehensively simulate the vertical distribution of VOCs.

In terms of radical chemistry and O_3_ formation mechanisms, Xue et al. analyzed radical budgets and O_3_ production using observations and the Master Chemical Mechanism (MCM) box model in Xianghe, a suburban site on the North China Plain (NCP), during summer 2018. The VOC composition was dominated by alkanes (62.2% of total VOCs), followed by equal contributions from alkenes and aromatics (17.3% each), with isoprene accounting for only 3.2%. Diurnal variations in NO_x_, CO, and AVOCs showed morning peaks synchronized with rush-hour traffic emissions, afternoon dilution due to BL elevation (>1.5 km), and nocturnal accumulation under shallow nighttime BL (<300 m). Isoprene peaked at ~1.8 ppbv (15:00 local time), decoupled from AVOCs, confirming its biogenic origin. O_3_ concentrations substantially exceeded standards, exhibiting a typical photochemical pollution pattern with afternoon maxima. HONO concentrations correlated positively with NO_2_, and box model results indicated that HONO photolysis contributed 41% to daytime RO_x_ production, while the reaction of NO_2_ with OH was the dominant radical termination pathway (accounting for 41%). Meanwhile, a cycle exists among RO_x_ radicals: OH acts as an atmospheric oxidant to promote the formation of HO_2_ and RO_2_, and the reactions of HO_2_ and RO_2_ with NO achieve rapid regeneration of OH. Sensitivity tests show that HONO is responsible for 42% of the O_3_ production in the simulations, as the cycling of RO_x_ radicals is linked to the production and depletion of O_3_. O_3_-NO_2_-VOC sensitivity analysis confirmed VOC-limited O_3_ production in Xianghe. When the absorption of aerosols on trace gases and radicals is taken into account, O_3_ generation is still in the VOC-controlled region, although it exhibits a trend of shifting towards NO_x_ sensitivity. This indicates that controlling VOCs is the optimal approach for alleviating O_3_ pollution in suburban NCP. Aerosol uptake of HO_2_ accounted for 11% of RO_x_ loss; neglecting this process would lead to O_3_ overestimation [27].

To sum up, future research should combine more advanced measurement technologies and models to comprehensively evaluate the characteristics of VOCs and photochemical smog in suburban areas and develop more effective pollution control strategies.

### 3.3. Rural Areas

During severe air pollution episodes on the NCP, organic aerosol (OA) constitutes a major pollutant. Biomass burning during wheat harvest periods has been identified as a primary source [57]. Organic nitrates (ONs), which are important SOA components [58], form via daytime OH oxidation of hydrocarbons in the presence of NO_x_ and nighttime NO_3_-initiated alkene oxidation. In rural NCP, particle-phase ONs are mainly associated with primary emissions, likely from biomass burning.

Despite severe aerosol pollution on the NCP, rural studies remain limited. Zhu et al. conducted offline PM_1_ measurements using thermal desorption–aerosol mass spectrometry (TD-AMS) in rural NCP. Positive matrix factorization (PMF) resolved four OA factors, hydrocarbon-like OA (HOA), biomass burning OA (BBOA), less-oxidized oxygenated OA (LO-OOA), and more-oxidized oxygenated OA (MO-OOA), with decreasing volatility (HOA > BBOA > LO-OOA > MO-OOA) and increasing O:C ratios. BBOA is an important component of OA, with an average contribution rate of 29.4%—second to LO-OOA (30.8%) and significantly higher than OA emitted from traffic sources (HOA, 18.4%) [57].

Ma et al. [59] reported that total BVOC emissions in low-altitude regions of Nanling were approximately double those at high-altitude sites, yet biogenic SOA (BSOA) concentrations were significantly higher at elevated locations. This suggests that environmental factors beyond BVOC precursors play critical roles in BSOA formation. At mountain summits, daytime SOA and O_3_ co-formation was driven by OH oxidation, while nocturnal SOA correlated with O_3_ oxidation of VOCs. In contrast, valley SOA levels correlated with NO_2_. BSOA tracers at both sites exhibited positive correlations with anthropogenic markers (NO_2_, SO_2_), indicating combined influences from biogenic and anthropogenic emissions. Similar findings in the Yangtze River Delta highlighted sulfate-enhanced BSOA formation [60].

In rural areas with minimal industrial influence (e.g., farmlands, forests), amines warrant attention. A concentration of ON ranging from 1.48 to 3.39 μg m^−3^ (8.1–19% of OA mass) exhibited strong correlations (r ≈ 0.7) with primary BBOA and black carbon from biomass burning (BC_bb), and its diurnal variation pattern was highly consistent with biomass burning activities, providing strong evidence that particulate-phase ON is primarily associated with the initial emissions from biomass burning [57]. In rural areas of the Yangtze River Delta (YRD), amines show a significant positive correlation with levoglucosan, which indicates biomass burning, suggesting that biomass burning is an important source of SOA precursors such as amines. Gas-phase amines, oxidized by O_3_ and OH, play key roles in aerosol formation and transformation processes. Particle-phase amines enhance aerosol stability via acid–base reactions or contribute to SOA through heterogeneous pathways [61].

Primary emissions from biomass burning are the dominant sources of OA and ON (contributing approximately 30%), whose SOA formation is highly sensitive to biomass burning events in rural NCP. In contrast, in the rural forests of YRD (Nanling), BSOA formation is more sensitive to anthro-biogenic source interactions, with BSOA concentrations being abnormally high (~150 ng m^−3^). Assessments indicate that controlling biomass burning alone may be insufficient to mitigate SOA pollution in rural areas, particularly when transboundary migration of anthropogenic pollutants (SO_2_, NO_x_, etc.) occurs. Developing differentiated precursor control strategies that are tailored to the characteristics of different rural environments (e.g., biomass burning-dominated regions versus regions influenced by anthropogenic pollution) is crucial for effectively alleviating SOA pollution. For example, to achieve coordinated regulation and control of SO_2_/NO_x_ and NH_3_, future models and observational studies should more precisely quantify the sensitivity coefficients of these rural characteristic parameters to key assumptions.

## 4. Modeling Studies on Atmospheric Oxidizing Capacity

### 4.1. Chemical Transport Models (CTMs): Core Tools for Research

#### 4.1.1. Key Features of WRF-Chem and CMAQ

**Figure 2:**
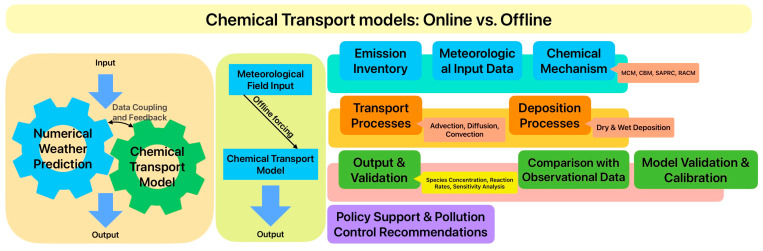
Principal operational process of online and offline CTMs.

Chemical transport models (CTMs) such as the Weather Research Forecasting Model with Chemistry (WRF-Chem) and Community Multiscale Air Quality model (CMAQ) are widely used to study atmospheric oxidizing capacity and SOA [62]. These models simulate the transport, diffusion, and chemical transformation of atmospheric aerosols and their precursors (e.g., O_3_, NO_x_, and PM_2.5_) while assessing the environmental effects of different emission control scenarios. Figure 2 shows the key features of CTMs.

Both WRF-Chem and CMAQ are typical regional-scale models, but they differ in their coupling frameworks, leading to distinct application scenarios. WRF-Chem, jointly developed by the U.S. National Oceanic and Atmospheric Administration (NOAA) and the National Center for Atmospheric Research (NCAR), employs an online-coupled framework, where meteorological processes and chemical reactions are solved simultaneously within a unified model core. This tight coupling minimizes temporal and spatial discontinuities, enabling accurate simulations of feedback mechanisms between meteorology and chemistry. WRF-Chem has demonstrated robust performance in simulating air pollutants and aerosols and is applicable to the study of aerosol–meteorology feedback and climate change driven by variations in chemical emissions [45,54]. Developed by the U.S. Environmental Protection Agency (USEPA), CMAQ focuses on simulating complex processes of secondary pollutants (e.g., O_3_, SOA). The offline modeling approach neglects the feedback mechanisms of atmospheric pollutants on meteorological processes, potentially introducing systematic biases into forecast outcomes. It operates offline, relying on external meteorological models (e.g., WRF) to drive chemical simulations [63].

#### 4.1.2. Case Studies: Applications of CTMs in Oxidation and SOA Research

Feng et al. [8] employed a modified WRF-Chem model to study autumn pollution events in Beijing, with configurations including a flexible gas-phase chemistry module and the CMAQ aerosol module [64]. Wet deposition followed the CMAQ scheme, dry deposition adopted the Wesely [65] parameterization, and photolysis rates were computed via the Fast-J module [66,67], accounting for cloud and aerosol effects. Inorganic aerosols were simulated using ISORROPIA v1.7 [68], which thermodynamically equilibrates NH_3_/NH_4_^+^, SO_4_^2-^, NO_3_^-^, Cl^-^, Na^+^, Ca^2+^, K^+^, Mg^2+^, and H_2_O, while SOA formation was modeled with the volatility basis set (VBS) approach [30,69,70]. Cai et al. used WRF-driven CMAQ to simulate July 2018 conditions, evaluating BVOCs’ impacts on O_3_ and SOA [69,70]. Validated against observations, the model showed acceptable statistical performance metrics. In numerical simulations of atmospheric environments, commonly used statistical test indicators for evaluating the accuracy of simulation results include the correlation coefficient (R), coefficient of determination (R^2^), normalized mean bias (NMB), normalized mean error (NME), mean bias (MB), mean absolute error (MAE), and root mean square error (RMSE) [62,71].

In a case study simulating the atmospheric pollution conditions in October 2014, the simulation of OH reactivity using the SAPRC07 mechanism yields the best results, with the simulated values being closest to the observed values (R^2^ = 0.83); the SAPRC99 mechanism follows, with an R^2^ value of 0.81. However, the SAPRC07 mechanism significantly underestimates the OH concentration on sunny days. Regarding the ozone production rate (PO_3_), RACM2 is closest to the observations during pollution periods, while MCMv3.2 and SAPRC99 perform well during clean periods [40].

Inherent uncertainties in numerical simulations of atmospheric processes predominantly originate from two aspects: the construction of input datasets and the characterization parameterization schemes within models. Specific sources encompass inaccuracies in meteorological field input datasets and pollutant emission inventories, uncertainties associated with VOC speciation, and limitations of physical parameterization schemes and chemical mechanisms (e.g., gas-phase chemical reactions, photolysis rate computations, and dry/wet deposition processes) [45]. These uncertain factors exert a significant impact on the simulation of meteorological conditions, the characterization of aerosol–radiation interactions, and the accuracy of assessments regarding the concentration and composition of particulate matter.

### 4.2. Machine Learning Models

Physicochemical models often face computational inefficiency, while purely data-driven models lack interpretability. Hybrid approaches combining both can improve pollution control strategies. Dong et al. [72] developed a correlation–ML-SHAP framework to analyze O_3_ drivers: correlation screening identified key factors, machine learning (XGBoost outperformed others) modeled relationships, and SHAP quantified factor contributions. Temperature (32.1%), solar radiation (21.3%), humidity (16.5%), and precursor emissions (15.6%) were dominant, with nonlinear interactions. This demonstrates machine learning’s utility in handling complex, nonlinear data. Future studies could integrate field/lab data for parameterization optimization. However, machine learning requires large datasets, and embedding physicochemical principles remains challenging. Although Dong et al.’s study quantified the driving effects of factors such as temperature and solar radiation on ozone, the machine learning model may have learned spurious correlations among environmental variables rather than true causal mechanisms. The lack of transparency or interpretability raises concerns about the reliability of model predictions when applied to extreme weather events or emission scenarios outside the training data distribution and makes it difficult to diagnose the physical causes of prediction errors. Traditional numerical models, despite their slowness, provide a self-consistent, physically consistent evolution of three-dimensional fields. In pursuit of inference speed, deep learning often employs simplified network architectures or down-scaled input features, which may lead to the model losing the ability to capture key microphysical processes.

Deep learning excels at processing complex data. Wang et al. [73] replaced the CBM-Z gas-phase mechanism in GNAQPMS with a ResNet model to predict radical concentrations. The model matched CBM-Z’s accuracy (mean *R*^2^ = 0.95) for OH, O_3_, and NO_x_ while being 300–750 times faster. Qiu et al. [74] combined numerical weather prediction (NWP) and deep learning in the PPN (PM_2.5_ Prediction Network) model for high-efficiency PM_2.5_ forecasting. PPN’s encoder–decoder architecture used historical PM_2.5_ and NWP fields to construct an initial state (analogous to CTM spin-up), with separate network layers for local (chemistry/turbulence) and nonlocal (transport) processes. A weighted loss function improved extreme PM_2.5_ event predictions. Applied to Beijing–Tianjin–Hebei (9 km resolution) in January 2022, the PPN achieved sub-second forecasts for 3-day predictions, outperforming WRF-Chem in accuracy and speed. However, the superior performance of the PPN model in the Beijing–Tianjin–Hebei region may be partly attributable to the dense monitoring network and long-term historical data accumulation in this area. If the same model were directly transferred to regions with sparse monitoring or entirely different climatic characteristics (such as oceanic or remote mountainous areas), its performance could degrade significantly. While deep learning models (e.g., ResNet and PPN) demonstrate superior computational efficiency and predictive accuracy compared with traditional numerical models (e.g., CBM-Z and WRF-Chem), this performance advantage often comes at the cost of model interpretability.

## 5. Limitations of Current Studies

### 5.1. Insufficient Observational Data and Uncertainties in Model Simulations

Current observations primarily focus on conventional species such as O_3_, NO_x_, and VOCs, while direct measurements of key atmospheric oxidants like OH, HO_2_, and RO_2_ remain relatively limited. Notably, the spatial distribution of existing monitoring sites exhibits significant heterogeneity: over 80% of these sites are concentrated in urban areas, while observational data from suburban regions, rural areas, and ecologically sensitive zones (e.g., forest ecosystems, coastal regions) are severely lacking, with the monitoring station density in these latter areas being less than 0.1 per 1000 km^2^.

Accurately assessing long-term trends in atmospheric oxidation capacity hinges on sustained and continuous observational datasets, which serve as robust constraints for numerical models. However, prevailing measurement techniques still face challenges in the accurate quantification of certain critical atmospheric oxidants and short-lived intermediates—particularly highly reactive, low-concentration radicals. Current direct measurement techniques for RO_x_ radicals remain immature. This limitation might be a significant factor constraining the performance of CTM simulations. For instance, measurements of RO_2_ are constrained by inherent limitations in instrumental sensitivity and selectivity issues; this technical bottleneck directly restricts our mechanistic understanding of key atmospheric oxidation processes. Addressing this gap would require sustained, in-depth scientific research to effectively bridge actual observations with model simulations. First, coordinated enhanced observations, such as comprehensive field campaigns, should be conducted to provide critical constraints for direct RO_x_ measurements, while advancing comparative studies of emerging measurement techniques. Second, a tiered validation framework is recommended for evaluating RO_2_ isomerization and POA oxidation mechanisms: mechanisms should first be validated using photochemical box model simulations with specific VOCs/POA and subsequently integrated into three-dimensional chemical transport models to examine their regional impacts. Finally, high-resolution or process-oriented modeling approaches should be applied to quantify the uncertainties in climate and air quality predictions resulting from aerosol–cloud–radiation interactions, thereby guiding the prioritization of parameterization improvements. Together, these steps will significantly enhance both the mechanistic realism and predictive reliability of chemical transport models. Furthermore, the precision of several oxidation-related indicators requires further refinement, and their temporal and spatial resolution remains insufficient to capture fine-scale environmental variations. The paucity of high-frequency, continuous observations hinders the detection of rapid short-term variations (e.g., NO_3_ burst events) and fine-scale spatial distribution patterns, as well as long-term (decadal or longer) trend analysis, undermining the reliability of future projections of atmospheric oxidation capacity [2]. A representative illustration of such technical constraints is the underestimation of organic amines’ contribution to SOA formation in rural North China: conventional analytical techniques (e.g., aerosol mass spectrometry) lack the capability to effectively distinguish organic amines from inorganic sulfates, leading to biases in source attribution and process quantification [75].

The atmospheric oxidation capacity is subject to non-negligible biases and uncertainties in numerical simulations. A typical example is the inaccurate simulation of tropospheric O_3_ formation in complex urban environments—a key limitation that undermines the model’s utility for urban air quality management. This bias is primarily attributed to the incomplete representation of critical atmospheric oxidation pathways in current chemical mechanisms. For instance, the omission of isomerization reactions involving RO_2_ has been identified as a major contributor: such reactions modulate the efficiency of NO_x_-dependent O_3_ formation, and their exclusion directly leads to systematic deviations between simulated and observed O_3_ concentrations.

Beyond gas-phase processes, the intricate reaction pathways between atmospheric oxidants (e.g., OH, O_3_, NO_3_) and POA remain insufficiently characterized. The lack of quantitative understanding of POA oxidation kinetics—including the formation of secondary organic aerosols (SOAs) and the evolution of aerosol chemical composition—hinders the seamless integration of these processes into numerical models, further amplifying uncertainties in the simulation of oxidation capacity.

Current models also exhibit notable deficiencies in parameterizing aerosol–cloud–radiation (ACR) interactions. Aerosols act as both participants and modulators of photochemical reactions: by scattering and absorbing solar radiation, they alter the photolysis rates of key precursors (e.g., NO_2_, H_2_O_2_) that drive oxidation reactions. Clouds introduce even greater complexity, as they not only scavenge aerosols and trace gases but also create aqueous-phase reaction environments (e.g., cloud droplets) that compete with or supplement gas-phase oxidation pathways. Under climate change and carbon neutrality strategies, the feedback between radiative forcing changes and atmospheric oxidation capacity has emerged as an urgent research priority. Alterations in temperature, solar radiation, and atmospheric circulation driven by climate change are expected to perturb the sources, sinks, and reaction rates of atmospheric oxidants, yet the magnitude and direction of these impacts remain poorly constrained by existing models, requiring targeted observational and modeling efforts. Moreover, the accuracy of oxidation capacity simulations is strongly limited by input datasets, particularly emission inventories and meteorological fields. Anthropogenic emission inventories covering key sectors such as industry, transportation, power generation, residential combustion, and agriculture exhibit significant uncertainties in both spatial distribution and emission intensity, stemming from incomplete activity data and uncertain emission factors. Biogenic emissions are equally uncertain, as they are highly sensitive to environmental conditions (e.g., temperature, solar radiation) that are themselves challenging to parameterize accurately in meteorological models.

### 5.2. Imperfect Chemical Mechanisms

SOA derived from photochemical oxidation of VOCs constitutes a major fraction of fine particulate matter in the troposphere, with profound implications for air quality and climate. However, the extreme complexity of photochemical reactions leaves many pathways and mechanisms unresolved, while the scarcity of kinetic data introduces uncertainties in SOA formation mechanisms. Multicomponent VOC mixtures under varying oxidation conditions can exhibit nonlinear SOA yields, where observed values significantly deviate from linear predictions based on individual precursors [76]. Current models systematically underestimate global SOA production. Small α-dicarbonyls (e.g., glyoxal and methylglyoxal), which are ubiquitous in the atmosphere and derived from aromatic VOC oxidation and biogenic isoprene, demonstrate conflicting experimental and theoretical contributions to SOA, particularly for methylglyoxal [77]. Wang et al. investigated methacrolein (MACR) photooxidation in Fe(III)–oxalate systems, revealing that oxalate markedly enhances MACR oxidation rates, modulated by Fe(III) concentration, initial MACR levels, and pH. The reaction yields low-volatility organic acids and high-molecular-weight oligomers, promoting SOA formation. Dynamic changes in solution absorbance further suggest potential impacts on aerosol’s optical properties and radiative forcing [78]. While SO_2_ generally promotes SOA via H_2_SO_4_-driven new particle formation (NPF) and acid-catalyzed carbonyl reactions, it may suppress SOA under specific conditions. For example, SO_2_ can scavenge OH and stabilized Criegee intermediates (sCIs), thus offsetting its oxidative role [7]. Direct SO_2_–peroxide interactions or other unexplored mechanisms may also exist. For instance, during particulate pollution episodes in the Beijing–Tianjin–Hebei region, NO_2_-catalyzed oxidation of SO_2_ represents the dominant sulfate formation pathway. Critically, Wang et al. [79] demonstrated that anion accumulation at aerosol gas–liquid interfaces significantly enhances NO_2_ uptake, accelerating SO_2_ oxidation and sulfate production while simultaneously generating gaseous HONO, which is a crucial OH precursor [80]. The current models significantly underestimate HONO concentrations (e.g., the NCAR Master Mechanism shows an underestimation of approximately 90%) due to the absence of key heterogeneous reactions in the chemical mechanism. Given that HONO is crucial for daytime RO_x_ and O_3_ production (contributing 55% and 42%, respectively) [27], this limitation leads directly to biases in the simulation of related photochemical processes. Such interface-enhanced oxidation effects should be incorporated into fundamental atmospheric chemical mechanisms and models. NH_3_ enhances SOA, primarily through gas-to-particle conversion of organic acids, although its efficiency depends on the BVOC precursors and oxidant types. Excess NH_3_ may decompose nascent SOA, while NH_3_–sCI reactions inhibit alternative SOA pathways. Acid-catalyzed NH_3_–carbonyl reactions in particles facilitate nitrogen-containing organic compounds (NOCs) and may enhance light absorption. Amine reactions with biogenic epoxides/carbonyls could modify the composition of SOA, although these processes require further study [7]. Systematic observations of amines across seasons and environmental conditions (e.g., O_3_, RH) remain lacking, obscuring their pollution levels, size distributions, and key drivers [61].

In summary, atmospheric oxidation involves highly complex chemistry. Despite progress, critical gaps persist, relating to questions such as competing pathways and transient intermediates in multi-step organic oxidations. Current mechanisms, often derived from idealized laboratory conditions, may not capture real-world complexity (e.g., high humidity/pollution). Future work must prioritize realistic environmental factors to resolve mechanisms of SOA formation and their relative contributions.

### 5.3. Key Knowledge Gaps

The lack of free radical measurements (especially RO_2_) and the imperfection of multiphase chemical mechanisms are the two most prominent bottlenecks leading to biases in AOC simulations. Despite significant progress in atmospheric chemistry research, several key knowledge gaps remain to be addressed. The current limitations in radical measurements stem from inadequate RO_2_ detection methods and insufficient vertical profiling capabilities. Our understanding of multiphase chemistry is hampered by incomplete parameterizations of critical aerosol–surface reactions, including SO_2_ uptake and NH_3_–organic acid partitioning processes. Model deficiencies persist in accurately representing RO_2_ isomerization, aerosol–radiation coupling, and emission uncertainties. Furthermore, the field requires that more mechanistic studies are conducted under realistic atmospheric conditions, particularly involving high-humidity and high-pollution scenarios. To overcome these challenges, future research should adopt an integrated approach combining advanced observational techniques (e.g., quantum cascade lasers for radical detection), controlled laboratory experiments, and machine learning-enhanced modeling frameworks. This multifaceted strategy will be essential for advancing our understanding of complex atmospheric processes.

## 6. Conclusions and Recommendations

The synergistic pollution of PM_2.5_ and ozone, driven by the AOC, remains a complex scientific and regulatory challenge. Despite advances through multi-platform observations and modeling, critical knowledge gaps hinder accurate prediction and effective control. These gaps are rooted in three interconnected limitations: persistent observational constraints, incomplete chemical mechanisms, and deficiencies in model representation.

To bridge these gaps, future research must adopt an integrated approach that systematically connects enhanced observations, refined mechanisms, and improved models. Within this framework, developing advanced methodologies to quantify key atmospheric processes emerges as an immediate priority. Specifically, the development of in situ free radical observation techniques is essential to overcome the current scarcity of direct radical measurements, which is a fundamental observational blind spot that limits our understanding of oxidation chemistry. Concurrently, improving the parameterization of gas–particle interface reactions is critical to resolve uncertainties in heterogeneous chemical pathways and to accurately represent aerosol–meteorology feedback in models, thereby reducing simulation biases for secondary aerosols.

These targeted efforts should be coordinated with broader initiatives to strengthen multidimensional monitoring networks, advance fundamental studies on VOC oxidation and multiphase chemistry, and refine regional emission control strategies. Ultimately, the convergence of these research streams with a focused investment in radical detection and interfacial process parameterization is vital to build robust predictive capabilities and support precision air quality management under carbon neutrality goals.

## Figures and Tables

**Table 1:** Seven fundamental equations for photochemical smog formation.

Step in Photochemical Smog Formation	Reaction Equations
NO_2_ absorbs UV radiation (300–400 nm) O_3_ reacts with NO	${NO}_{2}+hv\overset{k_{1}}{\to }NO+O$ $O+O_{2}+M\overset{k_{2}}{\to }O_{3}+M$ $O_{3}+NO\overset{k_{3}}{\to }{NO}_{2}+O_{2}$
Reactive hydrocarbons initiate chain reactions, forming radicals and products such as formaldehyde, acrolein, and peroxyacetyl nitrate (PAN)	$RH+O\overset{k_{4}}{\to }R\cdot +products$ $RH+O_{3}\overset{k_{5}}{\to }products(including\;R\cdot )$ $NO+R\cdot \overset{k_{6}}{\to }{NO}_{2}+R\cdot$
Radical termination	${NO}_{2}+R\cdot \overset{k_{7}}{\to }products(including\;PAN)$

## Data Availability

No new data were created or analyzed in this study. Data sharing is not applicable to this article.

## Supplementary Materials

The following supporting information can be downloaded at: https://www.mdpi.com/article/10.3390/toxics14020159/s1, Table S1: Key characteristics and parameters of atmospheric oxidation under different environmental (urban, suburban, rural) atmospheric pollutant emission backgrounds [81,82,83,84,85,86,87,88,89,90,91,92,93,94,95,96,97,98].
