# Supplementary material for: Advances and Challenges in Understanding Atmospheric Oxidizing Capacity in China: Insights from Chemical Mechanisms and Model Applications
Source: Toxics. 2026 Feb 8;14(2):159. doi: 10.3390/toxics14020159 (PMC12944996; doi:10.3390/toxics14020159)
Supplement: Supplementary file 1 [file toxics-14-00159-s001.zip › toxics-4094177-supplementary.pdf]

Supporting information for

Advances and Challenges in Understanding Atmospheric Oxidizing Capacity in China: Insights from Chemical Mechanisms and Model Applications

Peixuan Li[1], Yanqin Ren*[1], Fang Bi*[1], Fangyun Long[1], Junling Li[1], Haijie Zhang[1], Zhenhai Wu[1], Hong Li[1]

[1]State Key Laboratory of Environmental Criteria and Risk Assessment, Chinese Research Academy of Environmental Sciences, Beijing, 100012, China

Table S1. Key characteristics and parameters of atmospheric oxidation under different environmental (urban, suburban, rural) atmospheric pollutant emission backgrounds.

Type	Date	Location	O_3 (ppbv)	NO_2 (ppbv)	HONO (ppbv)	NH_3 (ppbv)	$PM_{2.5}$ (μg/m^3)	OC (μg/m^3)	EC (μg/m^3)	SOA (μg/m^3)	Ref.
Urban	2014	Beijing	55.9±37.0 (μg/m^3)	53.7±24.3 (μg/m^3)	/	/	99.6±76.5	/	/	/	[1]
	2014	Lanzhou	/	/	/	/	57±32	/	/	/	[2]
		Jinchang	/	/	/	/	45±35	/	/	/	
		Jiayuguan	/	/	/	/	34±30	/	/	/	
	2015	Shanghai	51.96±38.90	25.85±13.75	/	/	58.73±45.13	/	3.16±1.64	/	[3]
	2019		45.74±24.91	24.02±13.55	/	/	37.75±27.59	/	1.82±0.95	/	
	2021		47.28±22.71	22.35±15.60	/	/	34.27±25.48	/	1.50±0.94	/	
	Spring, 2017	Changzhou	43.5±28.5	22.9±14.9	1.55±1.21	/	47.9±25.2	/	/	/	[4]
	2017	Fujian	37.9-44.5	/	/	/	22.7-46.0	/	/	/	[5]
		Hunan	34.0-43.9	/	/	/	39.1-60.0	/	/	/	
	Oct-Dec, 2018	Beijing	/	32.23±7.43	2.52±1.61	/	100±69	/	/	/	[6]
	4 Dec, 2018-15 Mar, 2019	Xi’an	30.0±26.7	64.4±25.4	/	/	/	/	/	/	[7]
	Autumn, 2019 (mean)	Beijing	11.88	23.85	0.99	/	38.23	/	/	/	[8]
	Sep, 2019	Xiamen	67.4±17.2	15.4±6.9	/	/	/	/	/	/	[9]

Continued Table S1. Key characteristics and parameters of atmospheric oxidation under different environmental (urban, suburban, rural) atmospheric pollutant emission backgrounds.

Type	Date	Location	O_3 (ppbv)	NO_2 (ppbv)	HONO (ppbv)	NH_3 (ppbv)	$PM_{2.5}$ (μg/m^3)	OC (μg/m^3)	EC (μg/m^3)	SOA (μg/m^3)	Ref.
Urban	Spring, 2019	Ningde	28.17±15.86	11.94±6.22	/	/	/	/	/	/	[10]
	Summer, 2019		22.41±15.28	6.78±3.75	/	/	/	/	/	/	
	Autumn, 2019	Ningde	30.97±16.32	7.85±3.90	/	/	/	/	/	/	[10]
	Winter, 2019		18.44±13.44	12.30±6.44	/	/	/	/	/	/	
	11-13 Jun, 2019	Shanghai	65.13±27.16	14.20±6.13	0.36±0.16	/	/	/	/	/	[11]
	2-4 Sep, 2019		46.12±21.14	15.62±9.41	0.32±0.17	/	/	/	/	/	
	12-14 Jul, 2019		23.95±11.89	6.54±1.52	0.22±0.05	/	/	/	/	/	
	Apr-Dec, 2019	São Paulo, Brasil	/	/	/	/	27.6±9.9	5.50±3.42	1.68±1.61	/	[12]
		Medellín, Colombia	/	/	/	/	7.36±3.42	0.88±0.47	22.1±8.9	/	
	Apr, 2019-Mar, 2020	Quito, Ecuador	/	/	/	/	4.30±1.42	2.84±1.34	20.4±5.4	/	
		Ciudad de México, México	/	/	/	/	6.28±3.36	1.16±0.44	21.7±9.3	/	
	May,2019-Apr, 2020	San José, Costa Rica	/	/	/	/	5.07±1.83	2.89±1.17	27.1±9.3	/	

Continued Table S1. Key characteristics and parameters of atmospheric oxidation under different environmental (urban, suburban, rural) atmospheric pollutant emission backgrounds.

Type	Date	Location	O_3 (μg/m^3)	NO_2 (μg/m^3)	HONO (μg/m^3)	NH_3 (μg/m^3)	$PM_{2.5}$ (μg/m^3)	OC (μg/m^3)	EC (μg/m^3)	SOA (μg/m^3)	Ref.
Urban	2017/2018	Zhengzhou	36.7±28.9	56.8±28.4	2.5±2.4	12.4±8.1	95.7±78.9	12.2±7.4	3.1±2.3	/	[13]
	2018/2019		32.7±30.0	63.3±28.7	3.3±2.5	14.4±6.9	109.0±71.8	12.6±6.7	3.7±2.6	/	
	2019/2020		44.7±33.8	46.2±24.8	2.5±1.8	14.7±6.4	85.5±58.6	9.1±4.9	2.9±2.0	/	
	2020/2021		39.3±28.3	46.5±24.3	2.4±1.6	15.1±7.7	74.2±53.9	7.4±4.4	3.0±1.7	/	
	2021/2022		43.1±26.9	38.4±20.4	2.0±1.4	13.1±6.0	64.5±42.5	8.0±3.5	1.3±0.9	/	
	2022/2023		43.4±26.4	37.1±22.0	2.3±1.7	14.4±7.1	79.7±63.8	9.7±6.8	2.3±1.8	/	
	2018 (mean)	Beijing	/	/	/	/	33	/	/	3	[14]
		Tianjin	/	/	/	/	38	/	/	3	
		Shanghai	/	/	/	/	29	/	/	3	
		Nanjing	/	/	/	/	48	/	/	4	
		Hangzhou	/	/	/	/	37	/	/	4	
		Guangzhou	/	/	/	/	20	/	/	4	
		Dongguan	/	/	/	/	27	/	/	5	
		Shenzhen	/	/	/	/	22	/	/	4	
		Foshan	/	/	/	/	33	/	/	3	
		Wuhan	/	/	/	/	48	/	/	6	
		Xi’an	/	/	/	/	46	/	/	7	

Continued Table S1. Key characteristics and parameters of atmospheric oxidation under different environmental (urban, suburban, rural) atmospheric pollutant emission backgrounds.

Type	Date	Location	O_3 (ppbv)	NO_2 (ppbv)	HONO (ppbv)	NH_3 (ppbv)	$PM_{2.5}$ (μg/m^3)	OC (μg/m^3)	EC (μg/m^3)	SOA (ng/m^3)	Ref.
Suburban	2014	Jinchang	/	/	/	/	38±22	/	/	/	[2]
	Winter, 2015	Nanjing	10.45±6.80	23.92±7.84	1.32±0.92	/	114.19±59.59	/	/	/	[15]
	Summer, 2016	Nanjing	29.75±23.16	12.85±5.54	1.23±0.99	/	36.12±17.51	/	/	/	[15]
	Autumn, 2016		13.34±9.88	38.54±25.75	1.15±0.84	/	51.99±28.42	/	/	/	
	2017	Fujian	41.4-42.6	/	/	/	32.7-41.0	/	/	/	[5]
		Hunan	39.5-45.3	/	/	/	43.3-47.7	/	/	/	
	Summer, 2018	Xianghe	55.4±33.4	10.1±4.4	0.9±0.5	/	/	/	/	/	[16]
	Summer, 2019 (mean)	Xianghe	56.4	16.0	/	/	/	/	/	/	[17]
	Apr, 2019-Mar, 2020	Buenos Aires, Argentina	/	/	/	/	17.5±7.6	5.15±2.42	0.98±0.44	/	[12]
	Daytime, Jan, 2020	Xiamen	45.7±25.4 (μg/m^3)	33.0±8.50 (μg/m^3)	/	/	40.3±18.7	/	/	34.2±12.8	[18]
	Nighttime, Jan, 2020		37.6±16.8 (μg/m^3)	32.3±9.00 (μg/m^3)	/	/	45.1±17.0	/	/	40.4±19.6	

Continued Table S1. Key characteristics and parameters of atmospheric oxidation under different environmental (urban, suburban, rural) atmospheric pollutant emission backgrounds.

Type	Date	Location	O_3 (μg/m^3)	NO_2 (μg/m^3)	HONO (μg/m^3)	NH_3 (μg/m^3)	$PM_{2.5}$ (μg/m^3)	OC (μg/m^3)	EC (μg/m^3)	SOA (ng/m^3)	Ref.
Suburban	Daytime, Jul, 2020	Xiamen	80.3±46.2	12.2±6.50	/	/	19.4±9.70	/	/	158.3±102.5	[18]
	Nighttime, Jul, 2020		24.2±11.8	18.7±7.40	/	/	14.1±6.00	/	/	64.4±55.8	
	2017/2018	Baizhuang	22.1±21.5	47.4±21.5	3.15±2.71	19.3±10.6	117.4±93.4	28.1±22.0	6.3±5.1	/	[13]
	2018/2019		20.4±24.9	54.5±22.1	4.04±3.26	19.9±9.4	142.8±87.5	25.3±21.7	5.9±4.4	/	
	2019/2020		21.3±23.2	46.6±21.0	3.49±2.43	18.6±8.4	117.9±78.6	17.0±11.8	4.5±3.1	/	
	2020/2021		24.7±28.0	46.2±19.1	2.90±2.19	18.0±8.5	90.8±59.5	14.5±8.9	3.9±2.8	/	
	2021/2022		24.6±25.8	42.5±16.7	2.55±1.87	16.7±7.9	74.4±48.8	9.6±5.5	2.5±1.5	/	
	2022/2023		25.5±25.9	46.8±20.9	2.95±2.08	17.9±8.4	86.6±61.5	11.9±9.6	3.0±2.3	/	
Rural	2014	Lanzhou	/	/	/	/	51±23	/	/	/	[2]
		Jinchang	/	/	/	/	32±30	/	/	/	
	2017 (mean)	Wuyishan	50.8 (ppbv)	/	/	/	17.3	/	/	/	[5]
		Hengshan	48.7 (ppbv)	/	/	/	23.5	/	/	/	
		Nanling	46.9 (ppbv)	/	/	/	13.3	/	/	/	

Continued Table S1. Key characteristics and parameters of atmospheric oxidation under different environmental (urban, suburban, rural) atmospheric pollutant emission backgrounds.

Type	Date	Location	O_3 (μg/m^3)	NO_2 (μg/m^3)	HONO (μg/m^3)	NH_3 (μg/m^3)	$PM_{2.5}$ (μg/m^3)	OC (μg/m^3)	EC (μg/m^3)	SOA (μg/m^3)	Ref.
Rural	2017/2018	Wuzhi	28.9±21.2	38.3±21.6	2.7±2.5	27.7±15.3	106.5±85.5	15.9±12.3	4.4±3.5	/	[13]
	2018/2019		31.9±30.5	46.3±24.9	2.7±3.1	28.4±14.7	128.8±82.8	19.5±13.5	4.0±2.9	/	
	2019/2020		37.5±29.5	40.1±24.0	2.8±2.1	26.1±12.6	95.9±63.1	14.1±7.7	3.1±2.3	/	
	2020/2021		42.0±30.7	40.7±21.7	1.7±1.5	24.1±12.9	91.4±60.9	11.0±7.7	3.7±2.6	/	
	2021/2022		44.9±27.7	32.2±18.6	1.7±1.2	19.1±9.6	82.2±53.7	7.9±4.7	2.3±1.5	/	
	2022/2023		43.1±29.5	33.1±23.4	2.6±2.4	22.9±11.7	95.9±76.1	11.8±9.1	2.6±2.1	/	
	Autumn, 2018	Summit, Mt.Nanling	139.5±40.0	4.6±1.7	/	2.0±0.4	19.0±9.0	/	/	/	[19]
		Foot, Mt.Nanling	67.2±28.6	4.9±1.4	/	0.8±0.3	36.6±14.4	/	/	/	
	Spring, 2019	Bermuda	34.063±0.34 (ppbv)	0.17±0.092 (ppbv)	3.3±1.2 (pptv)	/	/	/	/	/	[20]
	Summer, 2019		13.43±0.62 (ppbv)	0.084±0.035 (ppbv)	3.2±1.2 (pptv)	/	/	/	/	/	

Continued Table S1. Key characteristics and parameters of atmospheric oxidation under different environmental (urban, suburban, rural) atmospheric pollutant emission backgrounds.

Type	Date	Location	O_3 (ppbv)	NO_2 (ppbv)	HONO (ppbv)	NH_3 (ppbv)	$PM_{2.5}$ (μg/m^3)	OC (μg/m^3)	EC (μg/m^3)	SOA (μg/m^3)	Ref.
Rural	Spring, 2019	Baengnyeong Island	44.03±10.71	3.16±2.23	/	/	/	/	/	/	[21]
	Summer,2019		45.74±17.29	4.10±2.97	/	5.07±2.17	/	/	/	/	
	Winter, 2019		35.35±6.61	5.56±4.87	/	1.53±1.08	/	/	/	/	
	Spring, 2020		51.18±9.83	1.93±2.35	/	3.67±1.70	/	/	/	/	
	Winter, 2020		27.06±7.46	4.76±3.55	/	2.93±2.98	/	/	/	/	
	Spring, 2021		36.64±10.20	4.69±2.49	/	3.22±1.09	/	/	/	/	
	Winter, 2021		29.26±5.34	4.31±3.00	/	2.35±2.32	/	/	/	/	

References

1. Wang, Y.-L.; Song, W.; Yang, W.; Sun, X.-C.; Tong, Y.-D.; Wang, X.-M.; Liu, C.-Q.; Bai, Z.-P.; Liu, X.-Y. Influences of Atmospheric Pollution on the Contributions of Major Oxidation Pathways to PM2.5 Nitrate Formation in Beijing. *Journal of Geophysical Research: Atmospheres* **2019**, *124*, 4174-4185, doi:https://doi.org/10.1029/2019JD030284.
2. Guan, Q.; Cai, A.; Wang, F.; Yang, L.; Xu, C.; Liu, Z. Spatio-temporal variability of particulate matter in the key part of Gansu Province, Western China. *Environmental Pollution* **2017**, *230*, 189-198, doi:https://doi.org/10.1016/j.envpol.2017.06.045.
3. Hu, H.; Liang, Y.; Li, T.; She, Y.; Wang, Y.; Yang, T.; Zhou, M.; Li, Z.; Li, C.; Xiao, H.; et al. Pathway-specific responses of isoprene-derived secondary organic aerosol formation to anthropogenic emission reductions in a megacity in eastern China. *EGUsphere* **2025**, *2025*, 1-34, doi:10.5194/egusphere-2025-1909.
4. Shi, X.; Ge, Y.; Zheng, J.; Ma, Y.; Ren, X.; Zhang, Y. Budget of nitrous acid and its impacts on atmospheric oxidative capacity at an urban site in the central Yangtze River Delta region of China. *Atmospheric Environment* **2020**, *238*, 117725, doi:https://doi.org/10.1016/j.atmosenv.2020.117725.
5. Gao, L.; Yue, X.; Meng, X.; Du, L.; Lei, Y.; Tian, C.; Qiu, L. Comparison of Ozone and PM2.5 Concentrations over Urban, Suburban, and Background Sites in China. *Advances in Atmospheric Sciences* **2020**, *37*, 1297-1309, doi:10.1007/s00376-020-0054-2.
6. Zhang, X.; Tong, S.; Jia, C.; Zhang, W.; Wang, Z.; Tang, G.; Hu, B.; Liu, Z.; Wang, L.; Zhao, P.; et al. Elucidating HONO formation mechanism and its essential contribution to OH during haze events. *npj Climate and Atmospheric Science* **2023**, *6*, 55, doi:10.1038/s41612-023-00371-w.
7. Duan, J.; Huang, R.J.; Gu, Y.; Lin, C.; Zhong, H.; Xu, W.; Liu, Q.; You, Y.; Ovadnevaite, J.; Ceburnis, D.; et al. Measurement report: Large contribution of biomass burning and aqueous-phase processes to the wintertime secondary organic aerosol formation in Xi'an, Northwest China. *Atmos. Chem. Phys.* **2022**, *22*, 10139-10153, doi:10.5194/acp-22-10139-2022.
8. Jia, C.; Tong, S.; Zhang, X.; Li, F.; Zhang, W.; Li, W.; Wang, Z.; Zhang, G.; Tang, G.; Liu, Z.; et al. Atmospheric oxidizing capacity in autumn Beijing: Analysis of the O3 and PM2.5 episodes based on observation-based model. *Journal of Environmental Sciences* **2023**, *124*, 557-569, doi:https://doi.org/10.1016/j.jes.2021.11.020.
9. Liu, T.; Hong, Y.; Li, M.; Xu, L.; Chen, J.; Bian, Y.; Yang, C.; Dan, Y.; Zhang, Y.; Xue, L.; et al. Atmospheric oxidation capacity and ozone pollution mechanism in a coastal city of southeastern China: analysis of a typical photochemical episode by an observation-based model. *Atmos. Chem. Phys.* **2022**, *22*, 2173-2190, doi:10.5194/acp-22-2173-2022.
10. Chen, G.; Liu, T.; Chen, J.; Xu, L.; Hu, B.; Yang, C.; Fan, X.; Li, M.; Hong, Y.; Ji, X.; et al. Atmospheric oxidation capacity and O3 formation in a coastal city of southeast China: Results from simulation based on four-season observation. *Journal of Environmental Sciences* **2024**, *136*, 68-80, doi:https://doi.org/10.1016/j.jes.2022.11.015.
11. Zhu, J.; Wang, S.; Wang, H.; Jing, S.; Lou, S.; Saiz-Lopez, A.; Zhou, B. Observationally constrained modeling of atmospheric oxidation capacity and photochemical reactivity in Shanghai, China. *Atmos. Chem. Phys.* **2020**, *20*, 1217-1232, doi:10.5194/acp-20-1217-2020.
12. Dawidowski, L.; Gelman Constantin, J.; Herrera Murillo, J.; Gómez-Marín, M.; Nogueira, T.; Blanco Jiménez, S.; Díaz-Suárez, V.; Baraldo Victorica, F.; Lichtig, P.; Díaz Resquin, M.; et al. Carbonaceous fraction in PM2.5 of six Latin American cities: Seasonal variations, sources and secondary organic carbon contribution. *Science of The Total Environment* **2024**, *948*, 174630, doi:https://doi.org/10.1016/j.scitotenv.2024.174630.
13. Ma, S.; Wang, N.; Zhang, J.; Ye, D.; Wang, L. Ammonia chemistry and oxidation dynamics as dual driving factors of PM2.5 nitrate pollution: Insights from the spatiotemporal disparities in central China. *Journal of Environmental Management* **2025**, *392*, 126594, doi:https://doi.org/10.1016/j.jenvman.2025.126594.
14. Azmi, S.; Sharma, M. Global PM2.5 and secondary organic aerosols (SOA) levels with sectorial contribution to anthropogenic and biogenic SOA formation. *Chemosphere* **2023**, *336*, 139195, doi:https://doi.org/10.1016/j.chemosphere.2023.139195.

15. Ge, Y.; Shi, X.; Ma, Y.; Zhang, W.; Ren, X.; Zheng, J.; Zhang, Y. Seasonality of nitrous acid near an industry zone in the Yangtze River Delta region of China: Formation mechanisms and contribution to the atmospheric oxidation capacity. *Atmospheric Environment* **2021**, *254*, 118420, doi:https://doi.org/10.1016/j.atmosenv.2021.118420.
16. Xue, M.; Ma, J.; Tang, G.; Tong, S.; Hu, B.; Zhang, X.; Li, X.; Wang, Y. ROx Budgets and O3 Formation during Summertime at Xianghe Suburban Site in the North China Plain. *AdAtS* **2021**, *38*, 1209-1222, doi:10.1007/s00376-021-0327-4.
17. Wang, R.; Wang, L.; Yang, Y.; Zhan, J.; Ji, D.; Hu, B.; Ling, Z.; Xue, M.; Zhao, S.; Yao, D.; et al. Comparative analysis for the impacts of VOC subgroups and atmospheric oxidation capacity on O3 based on different observation-based methods at a suburban site in the North China Plain. *Environmental Research* **2024**, *248*, 118250, doi:https://doi.org/10.1016/j.envres.2024.118250.
18. Hong, Y.; Xu, X.; Liao, D.; Liu, T.; Ji, X.; Xu, K.; Liao, C.; Wang, T.; Lin, C.; Chen, J. Measurement report: Effects of anthropogenic emissions and environmental factors on the formation of biogenic secondary organic aerosol (BSOA) in a coastal city of southeastern China. *Atmos. Chem. Phys.* **2022**, *22*, 7827-7841, doi:10.5194/acp-22-7827-2022.
19. Ma, F.; Wang, H.; Ding, Y.; Zhang, S.; Wu, G.; Li, Y.; Gong, D.; Ristovski, Z.; He, C.; Wang, B. Amplified Secondary Organic Aerosol Formation Induced by Anthropogenic–Biogenic Interactions in Forests Around Megacities. *Journal of Geophysical Research: Atmospheres* **2024**, *129*, e2024JD041679, doi:https://doi.org/10.1029/2024JD041679.
20. Elshorbany, Y.; Zhu, Y.; Wang, Y.; Zhou, X.; Sanderfield, S.; Ye, C.; Hayden, M.; Peters, A.J. Seasonal dependency of the atmospheric oxidizing capacity of the marine boundary layer of Bermuda. *Atmospheric Environment* **2022**, *289*, 119326, doi:https://doi.org/10.1016/j.atmosenv.2022.119326.
21. Kang, S.; Hong, S.; Lee, Y.; Park, G.; Park, T.; Ban, J.; Kim, K.; Kim, Y.; Choi, Y.; Park, J.; et al. Seasonal chemical characteristics and formation of potential secondary aerosols of a remote area in South Korea using an oxidation flow reactor. *Atmospheric Environment* **2025**, *355*, 121216, doi:https://doi.org/10.1016/j.atmosenv.2025.121216.
